# Proteasome inhibition alleviates prolonged moderate compression-induced muscle pathology

**DOI:** 10.1186/1471-2474-12-58

**Published:** 2011-03-07

**Authors:** Parco M Siu, Bee T Teng, Xiao M Pei, Eric W Tam

**Affiliations:** 1 Department of Health Technology and Informatics, The Hong Kong Polytechnic University, Hung Hom, Kowloon, Hong Kong, China

## Abstract

**Background:**

The molecular mechanism initiating deep pressure ulcer remains to be elucidated. The present study tested the hypothesis that the ubiquitin proteasome system is involved in the signalling mechanism in pressure-induced deep tissue injury.

**Methods:**

Adult Sprague Dawley rats were subjected to an experimental compression model to induce deep tissue injury. The tibialis region of the right hind limb was subjected to 100 mmHg of static pressure for six hours on each of two consecutive days. The compression pressure was continuously monitored by a three-axial force transducer within the compression indentor. The left hind limb served as the intra-animal control. Muscle tissues underneath the compressed region were collected and used for analyses.

**Results:**

Our results demonstrated that the activity of 20S proteasome and the protein abundance of ubiquitin and MAFbx/atrogin-1 were elevated in conjunction with pathohistological changes in the compressed muscle, as compared to control muscle. The administration of the proteasome inhibitor MG132 was found to be effective in ameliorating the development of pathological histology in compressed muscle. Furthermore, 20S proteasome activity and protein content of ubiquitin and MAFbx/atrogin-1 showed no apparent increase in the MG132-treated muscle following compression.

**Conclusion:**

Our data suggest that the ubiquitin proteasome system may play a role in the pathogenesis of pressure-induced deep tissue injury.

## Background

Pressure ulcer represents a considerable clinical problem. An estimated one billion dollars is spent annually to manage pressure ulcers in the United States [1]. A pressure ulcer is defined as localized damage to the skin and the underlying tissues in response to moderate but sustained mechanical compression [2]. The exact cause and pathogenesis of pressure ulcers are largely unknown. Pressure ulcers are generally categorized into superficial or deep pressure ulcers. These are distinctly differentiated based on their developmental processes. A superficial ulcer is confined to the skin layer, and can be diagnosed visually at an early stage. In contrast, a deep ulcer originates in the underlying tissues overlying a bony prominence in the compressed region, and its development involves subsequent progression of the injury upwards until it penetrates to the skin [3]. Deep ulcer is of considerable clinical concern as the tissue damage is already very severe by the time the ulceration becomes visible and detectable at the cutaneous layer. This makes the prognosis very uncertain and hinders therapeutic follow-up. To better characterize this serious form of pressure ulcer, the term "deep tissue injury" has been introduced by the US National Pressure Ulcer Advisory Panel (NPUAP) in order to emphasize that deep pressure ulcer occurs as "a pressure-related injury to subcutaneous tissues under intact skin" [4-9].

Ubiquitin proteasome system is a non-lysosomal cellular process responsible for the housekeeping functions of the regulation of protein turnover and the elimination of abnormal proteins in eukaryotic cells. This system has been shown to be involved in the regulation of important biological events including cell division, stress response [10], signalling transduction [11,12], transcription, and protein sorting [13]. Ubiquitin proteasome system could probably be one of the potential candidates of the molecular target responsible for the pathogenesis of pressure ulcer. In particular, the ubiquitin proteasome system signalling has been shown to be critical for coordinating muscle degradation [14]. In this pathway, proteins tagged with a polyubiquitin signal are degraded by a proteasome complex. Ubiquitination is the process of the covalent attachment of ubiquitin to a protein substrate. The formation of a polyubiquitin chain essentially relies on the E3 ligases which function to selectively recognize their specific protein substrates [15]. Muscle specific E3 ubiquitin ligases, including muscle atrophy F-box/atrophy gene-1 (MAFbx/atrogin-1) and muscle ring finger 1 (MuRF1), have been previously identified [16,17]. MAFbx/atrogin-1 contains an F-box domain which is a characteristic motif found in the SCF (Skp1, cdc53/cullin and F-box protein) subfamily of E3 ubiquitin ligases. The F-box functions to link the elements of the E3 complex to the targeted protein [15,18]. MuRF1 belongs to the RING Finger E3 ligase subfamily which was initially found in association with the myofibril [18,19]. These two muscle-specific E3 ligases have been demonstrated to be upregulated in muscle degradation due to disuse and to catabolic conditions. They are generally considered as the critical regulatory components of the ubiquitin-proteasome system mediated catabolic process [2,14,15,20].

Research has resulted in some but limited understanding of the etiology of pressure ulcer. The identified critical risk factors include shear, moisture, friction, malnutrition, and pressure [21,22]. Yet, the fundamental knowledge of the pathology of deep pressure ulcer is still missing. The cellular signalling events coordinating deep tissue injury need to be revealed so that novel preventive and treatment regimens can be developed. Driven by the fact that skeletal muscle tissue is particularly sensitive to prolonged compression [23-26] and the destructive nature of the ubiquitin proteasome system in muscle [18,27,28], the present study investigated whether the muscle ubiquitin proteasome system is involved in the underlying mechanism of the pathology of deep pressure ulcer. We tested the hypothesis that the muscle ubiquitin proteasome system plays a causative role in pressure-induced deep tissue injury. As the pathogenesis of deep pressure ulcer might involve the hypoxia- and cellular deformation-related mechanisms, the gene expression levels of molecular markers of hypoxia and cellular chaperone including hypoxia inducible factor (HIF-1α) and heat shock protein70 (HSP70) were also investigated in this study.

## Methods

### Animals

Female adult Sprague Dawley rats weighing about 300 g were used in this study. The animals were kept in conventional housing under pathogen-free conditions, exposed to a reverse light condition of 12:12-hours light-dark, and fed with standard nutrient diet and water ad libitum. Animal research ethics approval was obtained from the Animal Ethics Sub-committee of The Hong Kong Polytechnic University. The animal care standards were followed by adhering to the recommendations for the care of laboratory animals as advocated by the American Association for Accreditation of Laboratory Animal Care (AAALAC) and following the policies and procedures detailed in the Guide for the Care and Use of Laboratory Animals as published by the U.S. Dept. of Health and Human Services and proclaimed in the Animals Welfare Act.

### Muscle Compression Procedure

After acclimatization with the housing environment, rats (N = 8) were subjected to an in vivo pressure-induced deep tissue injury protocol, previously established in the same laboratory, with minor modifications [29-31]. Briefly, animals were first anesthetized with ketamine (80 mg/kg) and xylazine (8 mg/kg) by intra-peritoneal (i.p.) injection. One third of the initial dose was i.p. administered approximately one to two times throughout the compression procedure to maintain the level of anaesthesia. Anesthetization was assured by testing the loss of reflex action and moustache dithering test. The hind limbs were shaved with an electronic razor before being subjected to compression, which involved moderate prolonged compression loading with a static pressure of 100 mmHg. This was applied to an area of 1.5 cm^2 ^over the tibialis region of the right limb. The loading duration was six hours on each of two consecutive days. The compression force was continuously monitored by a three-axial force transducer within the compression indentor. A laser Doppler flowmetry (DRT4, Moor Instruments, Axminster, UK) with a contact probe (DP1T/7-V2) was used to monitor the blood flow of the compression site as previously described [29]. The left uncompressed limb served as intra-animal control. Rats were killed by overdose of ketamine and xylazine twenty hours after the last session of compression. Tissue samples were excised from the area directly underneath the indentor region, frozen in liquid nitrogen-cooled isopentane and stored at -80°C until further analysis. To examine the MAFbx/atrogin-1 protein expression in muscle immediately after the compression protocol, muscle samples of the rats that were sacrificed immediately after two sessions of 6-hours of compression over two consecutive days (2D-IM group) [31] were used to perform Western blot analysis of MAFbx/atrogin-1.

### Administration of Proteasome Inhibitor

Rats were randomly divided into two groups to receive either MG132 (carbobenzoxy-L-leucyl-L-leucyl-L-leucinal, Z-LLL-CHO) or DMSO (vehicle control) administration (N = 5 per group). MG132, a cell-permeable peptide aldehyde that inhibits the major peptidase activities of proteasome [32] was purchased from Merck and dissolved in DMSO for use. A dose of 10 mg/kg of MG132 that has been used in previous studies was adopted in this study [33,34]. The MG132 administration was carried out by two intraperitoneal injections of 5 mg/kg of MG132 right before each of the two 6-hours compression sessions. Equal volumes of DMSO were administered to the control animals.

### Histological Analysis

Standard hematoxylin and eosin staining procedure was used to examine the histology of tissues. Ten micro-meters thick cross-sections were prepared from frozen muscle tissue samples in a cryostat at -20°C. The sections were air dried at room temperature and fixed with 10% formalin solution (HT-5011, Sigma Aldrich). Samples were stained with Mayer's hematoxylin (MHS-1, Sigma Aldrich) and 1% eosin in CaCl_2 _(318906, Sigma Aldrich) following a series of alcohol-mediated dehydration steps, and stained sections were mounted in clarion mounting medium (C-0487, Sigma Aldrich).

### Real time Quantitative PCR Analysis

Total RNA was extracted from frozen compressed and control muscle samples with TriReagent (Molecular Research Center) based on the guanidine thiocynate method. Muscle samples were homogenized mechanically in ice-cold TriReagent. The extracted total RNA was solubilized in RNase-free H_2_O and quantified by spectrophotometry at λ = 260 nm and the purity of RNA was confirmed by examining the 260/280 ratio. Superscript III reverse transcriptase kit (Invitrogen Life Technology) was used to generate complementary DNA (cDNA). Reverse transcription was performed by following the manufacturer's recommendations, with a reaction volume of 20 μl which included 1 μg of total RNA, decamer primers and superscript reverse transcriptase. For the cDNA prepared from the preliminary samples collected from the rats which had not been treated with DMSO or MG132, the PCR reaction was performed in TaqMan Master Mix, primer and Taqman probes, and RNase and DNase free water on an ABI7500 real time PCR thermocycler (Applied Biosystems). Primers and probes for the TaqMan assay were designed specifically against the sequence of rat MAFbx/atrogin-1, MuRF1, and glyceraldehyde-3-phosphate dehydrogenase (GAPDH) by the manufacturer [MAFbx/atrogin-1: NM_133521.1 (GenBank accession number), Rn00591730_m1 (Applied Biosystems TaqMan assay ID), 61 bp (Amplicon length); MuRF-1: NM_080903.1, Rn00590197_m1, 56 bp; GAPDH: NM_017008.3, Rn99999916_s1, 87 bp]. For the TaqMan PCR, GAPDH was used as internal control and the relative quantification of gene expression was calculated using the comparative threshold cycle ΔΔC_T _method [35]. For the cDNA prepared from the samples collected from the rats which had been treated with DMSO or MG132, the PCR reaction was performed in SYBR green/ROX qPCR Master Mix (Fermentas) with forward and reverse primers for ubiquitin, MAFbx/atrogin-1, HIF-1α, HSP70 or β2 microglobulin (β2M) (Table 1), and RNase/DNase-free water in ABI7500 real time PCR thermocycler (Applied Biosystems). For the SYBR green/ROX-mediated PCR, β2M was included as internal housekeeping control gene. A relative standard curve (concentration vs. threshold cycle) of target and reference genes for quantification of PCR products was generated by dilution of cDNA from the calibrator. Complementary DNA prepared from uncompressed control samples was used as the calibrator to generate the standard curve. Results were expressed as the concentration ratio of the target gene to β2M of each sample. All PCR amplification and sequences were verified and the threshold for kinetic detection was set to occur over linear amplifications over several ranges of primers and RNA levels. The amplification efficiency for the PCR reactions was assessed by including serially diluted cDNAs with known DNA concentrations as positive control. All samples were run in duplicate, with control and compressed samples run on the same plate.

**Table 1:** Primer Used in Real Time RT-PCR Analysis

Gene	Accession Number	Primers
		(F; forward primer, R; reverse primer)
Ubiquitin	NM017314	F: 5'GGGCATGCAGATCTTTGTGAA3'
		R: 5'ACCTCCAGGGTGATGGTCTTG3'
MAFbx/atrogin-1	NM133521	F: 5'TGAAGACCGGCTACTGTGGAAGAGAC3'
		R: 5'TTGGGGTGAAAGTGAGACGGAGCAG3'
HIF-1α	NM024359	F: 5'AACAAACAGAATCTGTCCTCAAACC3'
		R: 5'CAGGTAATGGAGACATTGCCAG3'
HSP70-1	X77207	F: 5'CGAGGGCATCGACTTCTACACG3'
		R: 5'ATCTGCGCCTTGTCCAGCTTG3'
HSP70-2	X77208	F: 5'CTCGTCCATGGTGCTGACCAAG3'
		R: 5'CCGCTGCGAGTCGTTGAAGTAG3'
HSP70-3	X77209	F: 5'AGGACTCAACGTGCTGCGAATC3'
		R: 5'TCAGGATGGACACGTCGAACG3'
β2M	NM012512	F: 5'CGTGATCTTTCTGGTGCTTGTC3'
		R: 5'TTCTGAATGGCAAGCACGAC3'

### Proteasome Activity Assay

The proteasome enzymatic activity was measured by using a proteasome 20S assay kit (Enzo Life Sciences) following the manufacturer's instructions. In brief, the protein extract from muscle tissue was used to perform assessment of proteasome 20S activity by measuring the hydrolysis of a fluorogenic peptidyl substrate Suc-Leu-Leu-Val-Tyr-AMC (AMC: 7-amino-4-methylcoumarin) following the 0.03% sodium dodecyl sulphate-activation step. This substrate was cleaved by the proteasome activity and the subsequently released free AMC was then detected by a fluorimeter with an excitation wavelength of 380 nm and emission wavelength of 460 nm. The fluorescence signal was monitored before and after one-hour incubation at 37°C. The change in fluorescence signal was normalized to the amount of protein used in the assay.

### Immunocytochemical Analysis

Frozen tissue cross-sections were cut to a thickness of 10 μm in a cryostat at -20°C. The sections were air dried at room temperature and fixed with 10% formalin solution (Sigma Aldrich). Background activity was minimized by blocking the section with 5% goat serum. After washing, sections were incubated with anti-ubiquitin mouse monoclonal (3936, Cell Signaling), anti-MAFbx rabbit polyclonal (sc-33782, Santa Cruz Biotechnology) or anti-MuRF1 rabbit polyclonal (sc-32920, Santa Cruz Biotechnology) antibodies. Sections were then processed by using Vectastain Elite ABC Peroxidase kit (PK6100, Vector Laboratories) with ImmPACT DAB peroxidase substrate (SK4105, Vector Laboratories) or exposed to anti-rabbit IgG (H+L) fluorescein-conjugated secondary antibody (FI-1000, Vector Laboratories). Negative controls were performed by eliminating the primary or secondary antibody. Sections were examined under a Nikon 80i microscope. Images were captured with a Nikon DXM 1200C camera using Nikon ACT-1C software.

### Western Blot Analysis

Protein abundance of ubiquitin and MAFbx/atrogin-1 was measured in the compressed and control muscles. Soluble protein was extracted from muscle samples by the following procedure. After mincing and homogenization in ice-cold lysis buffer (10 mM NaCl, 1.5 mM MgCl_2_, 20 mM HEPES, pH 7.4, 20% glycerol, 0.1% Triton X-100, and 1 mM dithiothreitol), muscle homogenate underwent serial centrifugations. Protease inhibitor cocktail (P8340, Sigma-Aldrich) was added to the extracted protein followed by spectrophotometric measurement of protein concentration at 595 nm using a commercial Bradford method (Coomassie Protein Assay, Pierce). The protein concentration was determined in duplicate by following the manufacturer's recommendations with bovine serum albumin (BSA) used as standard. Protein extracts were then boiled at 95°C for 5 min in Laemmli buffer with 5% β-mercaptoethanol. Forty μg of protein was loaded on a 10% polyacrylamide gel. After electrophoretic separation by SDS-PAGE, the proteins were transferred to polyvinylidene difluoride (PVDF) membranes (Immobilon P, Millipore). The membranes were blocked in 5% nonfat milk in Tris-buffered saline with 0.1% Tween 20 (TBST) for 1 h at room temperature after the transfer, and incubated overnight at 4°C with corresponding primary antibody: anti-ubiquitin mouse monoclonal antibody (1:1000 dilution, 3936, Cell Signaling) or anti-MAFbx/atrogin-1 rabbit polyclonal antibody (1:200 dilution, sc33782, Santa Cruz) diluted in TBST with 2% bovine serum albumin (BSA). Membranes were then washed in TBST and incubated with horseradish peroxidase (HRP)-conjugated secondary antibodies at room temperature for 1 h (1:3000 dilution, 7076 for anti-mouse antibody, 7074 for anti-rabbit antibody, Cell Signaling). Luminol reagent (NEL103001EA, Perkin Elmer) for chemiluminescent detection of HRP was then applied. The chemiluminescent signal was captured with a Kodak 4000R Pro camera. The resulting bands were quantified as optical density (OD) x band area and expressed as arbitrary units. β-tubulin (1:2000 dilution, T0198, Sigma Aldrich) was probed and used as the reference of internal control. Data on ubiquitin and MAFbx/atrogin-1 were expressed by normalizing to the signal of β-tubulin.

### TUNEL Analysis

DNA strand breaks were assessed by fluorescent labelling of terminal dUTP nick end labelling (TUNEL) using a detection kit according to the manufacturer's instructions (11684795910, Roche Applied Science). In brief, 10 μm thick frozen sections were cut in a freezing cryostat at -20°C. Tissue sections were air dried at room temperature, fixed with 4% paraformaldehyde in PBS, permeabilized with 0.2% Triton X-100 in 0.1% sodium citrate, and incubated with the fluorescein-conjugated TUNEL reaction mixture. A section treated with DNase I was examined as positive control. Omission of the addition TdT enzyme in the TUNEL reaction mixture was included as negative control. The sections were then mounted with DAPI mounting medium to visualize nuclei (Vectashield mounting medium, Vector Laboratories). TUNEL- and DAPI-stained nuclei were examined under a fluorescence microscope (Biological Research Microscope 80i, Nikon) equipped with a digital camera (DXM 1200c, Nikon) using Nikon ACT-1C software. SPOT RT software (Diagnostic Instruments) was then used to stack and analyse the images.

### Statistical Analysis

All data are presented as mean ± standard error of mean. One-way ANOVA with Tukey HSD post hoc test was used to examine differences between groups. Level of statistical significance was set at p < 0.05.

## Results

### Compression induced muscle pathohistology and increase in MAFbx/atrogin-1 protein expression

**Figure 1:**
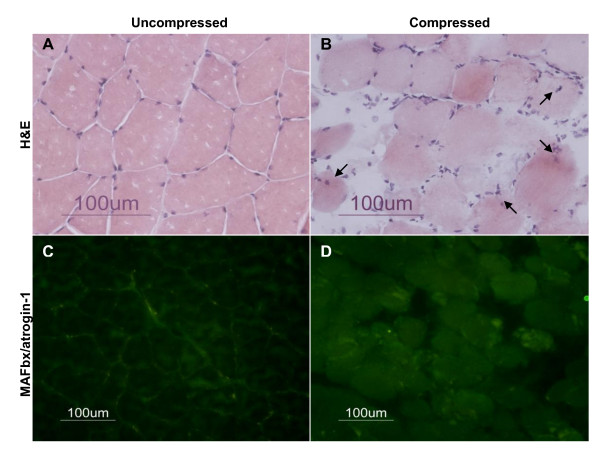
**Compression-induced Pathohistology and Increase in MAFbx/atrogin-1**. Preliminary experiments were conducted to expose untreated rats (neither DMSO nor MG132) to the compression protocol to examine the effect of compression on gross muscle histology and expression of ubiquitin E3 ligases, MAFbx/atrogin-1 and MuRF1. Gross muscle histology was examined following hematoxylin and eosin staining. Muscle tissue after compression generally showed pathohistological appearances including rounding shaped muscle cells, accumulated nuclei number, and internalization of peripherally located nuclei in muscle cells (as shown by arrows in B). Fluorescent immunocytochemical analysis demonstrated that the protein expression of MAFbx/atrogin-1 was apparently elevated in cytoplasmic region of the compressed muscle (D) relative to uncompressed muscle (C). No noticeable change in immunoreactivity of MuRF1 was found in the compressed and uncompressed muscles (data not shown).

Preliminary experiments were conducted in rats which had not been treated with DMSO or MG132 to demonstrate the potential involvement of the ubiquitin proteasome system in compression-induced muscle pathology. Gross muscle histology and expression of ubiquitin E3 ligases, MAFbx/atrogin-1 and MuRF1 were examined. Histological analysis demonstrated the presence of pathological muscle changes following compression (Figure 1 A and 1B). These included loss of angular shape and rounding contour of cross-sectional muscle fibres, increase in nuclei number in the interstitial space, increased proportion of interstitial space, and internalization of peripherally located nuclei in muscle cells (Figure 1 B). These pathohistological characteristics were noticeably in contrast to the normal histology of muscle cells in uncompressed samples which showed tightly packed muscle fibres with polygonal shape and peripherally located nuclei (Figure 1 A). According to our fluorescent immunocytochemical analysis, immunoreactivity of MuRF1 was found to be unchanged in muscle in response to compression. However, immunoreactivity of MAFbx/atrogin-1 localized in the cytoplasmic region of the compressed muscle was apparently increased when compared to uncompressed muscle (Figure 1 C and 1D). The mRNA expression of MAFbx/atrogin-1 and MuRF1 was also examined by real time quantitative PCR. No significant difference in mRNA content of MAFbx/atrogin-1 and MuRF1 was observed with compression treatment.

### MG132 treatment alleviated compression-induced muscle pathohistology

**Figure 2:**
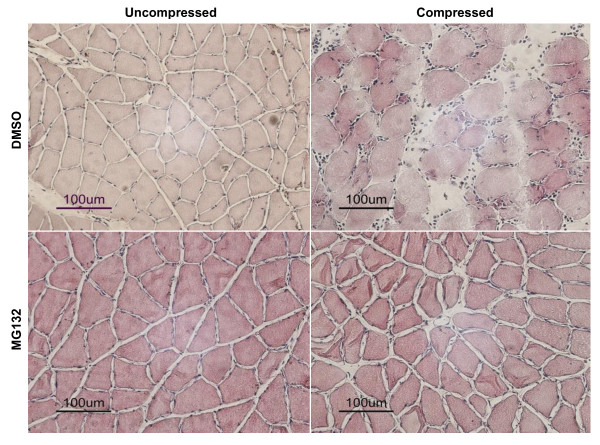
**Histological Analysis**. Muscle histology was examined following hematoxylin and eosin staining. Histologic appearances indicating muscle pathology such as loss of tightly packed polygonal muscle cells arrangement, round shaped contour of muscle cells, and increment of nuclei number were generally observed in compressed muscle of DMSO-treated animals but not MG132-treated animals.

The gross muscle histological changes as induced by compression in DMSO vehicle-treated rats were similar to that in the untreated rats. Histological analysis demonstrated that changes such as loss of tightly packed polygonal arrangement of muscle cells, round shaped contour of muscle cells, and increase in nuclei number were generally observed in compressed muscle of DMSO-treated animals (Figure 2 upper panels). In contrast, these pathohistological appearances were not seen in the muscle of MG132-treated animals following compression (Figure 2 lower panels).

### MG132 treatment precluded compression-induced elevation of 20S proteasome activity

**Figure 3:**
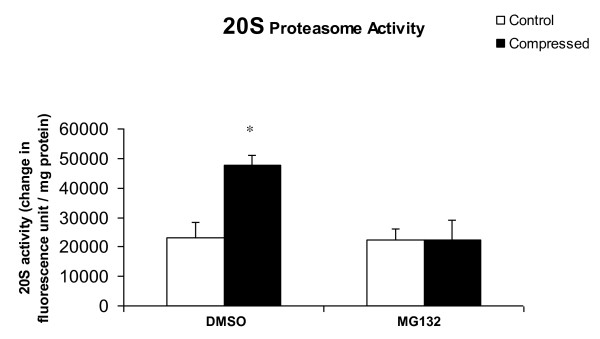
**20S Proteasome Activity Assay**. 20S proteasome activity was increased in the compressed muscle of DMSO-treated animals but not MG132-treated animals. Results were expressed as change in fluorescence unit normalized to the amount (milligrams) protein used in the assay. Data are presented as means ± SEM. *p < 0.05 compared with the uncompressed muscle of DMSO-treated animals.

The 20S proteasome activity in compressed muscle of DMSO-treated animals was significantly increased by 105% relative to the uncompressed muscle (Figure 3). In MG132-treated animals, no significant difference was found in 20S proteasome activity between compressed and uncompressed muscles (Figure 3).

### MG132 treatment precluded compression-induced increase in ubiquitin and MAFbx/atrogin-1

**Figure 4:**
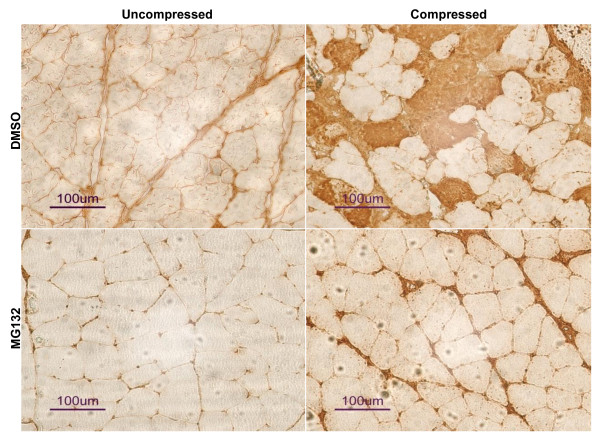
**Immunocytochemical Analysis of Ubiquitin**. DAB-mediated immunostaining was used to examine the ubiquitin protein abundance in uncompressed and compressed muscles of DMSO- and MG132-treated animals. In DMSO-treated rats, uncompressed muscle did not show any immunoreactivity of ubiquitin whereas compressed muscle demonstrated strong immunoreactivity in the muscle cells (upper panels). The ubiquitin immunoreactivity was distributed in muscle fibres in a scattered pattern. In MG132-treated rats, no immunoreactivity of ubiquitin was detected in both uncompressed and compressed muscles (lower panels).

**Figure 5:**
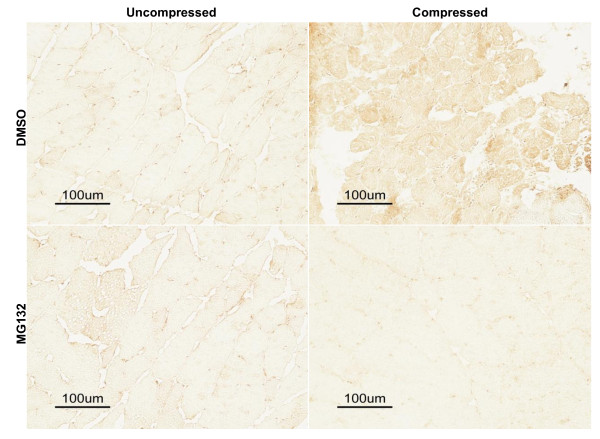
**Immunocytochemical Analysis of MAFbx/atrogin-1**. DAB-mediated immunocytochemistry was used to examine the protein abundance of MAFbx/atrogin-1 in uncompressed and compressed muscles of DMSO- and MG132-treated animals. Uncompressed muscle of both DMSO- and MG132-treated rats did not show any immunoreactivity of MAFbx/atrogin-1. Apparent positive immunoreactivity was detected in the muscle fibres of compressed muscle in only DMSO-treated rats but not in MG132-treated rats.

**Figure 6:**
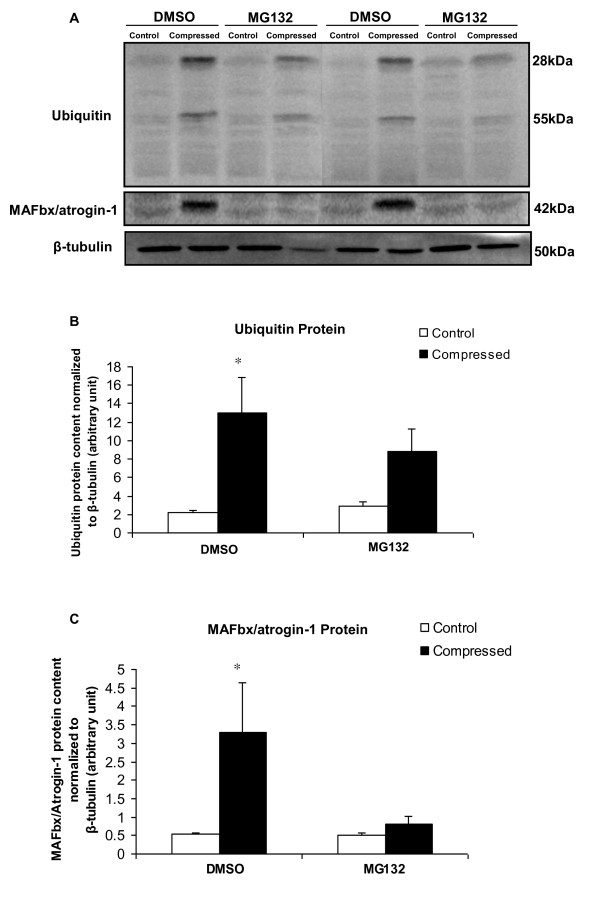
**Protein Expression of Ubiquitin and MAFbx/atrogin-1**. Panel A shows the representative Western immunblots of ubiquitin, MAFbx/atrogin-1, and β-tubulin in compressed and control muscles in two pairs of DMSO and MG132 treated animals. The protein expression of ubiquitin and MAFbx/atrogin-1 was determined by Western blot. Data are presented as net intensity x resulting band area and expressed in arbitrary units. Results of ubiquitin (B) and MAFbx/atrogin-1 (C) were normalized to corresponding β-tubulin signal.

The protein content of ubiquitin and MAFbx/atrogin-1 was examined by immunocytochemical analysis. Marked immunopositive staining of ubiquitin was found to be localized in the cytoplasmic region of scattered muscle fibres with round contours in the compressed muscle of DMSO-treated rats whereas no immunoreactivity of ubiquitin was detected in uncompressed muscle of DMSO-rats (Figure 4 upper panels). In contrast, immunoreactivity of ubiquitin was not detected in either uncompressed or compressed muscles of MG132-treated rats (Figure 4 lower panels). According to our immunocytochemical analysis, MAFbx/atrogin-1 protein abundance was apparently increased in the compressed muscle relative to uncompressed muscle of DMSO-rats (Figure 5 upper panels). However, MAFbx/atrogin-1 protein content was not changed in compressed muscle when compared to uncompressed muscle in MG132-treated rats (Figure 5 lower panels). MuRF1 protein abundance was also examined but no apparent change was found in the compressed muscle relative to uncompressed muscle in both DMSO- and MG132-treated animals (data not shown). According to our Western immunoblot analysis, the protein abundance of ubiquitin was significantly increased by 4.8-folds in the compressed muscle when compared to uncompressed muscle of DMSO-treated rats. The protein abundance of ubiquitin appeared to be increased in the compressed muscle relative to the uncompressed muscle in MG132-treated rats but this did not reach the statistical significance level (p > 0.05) (Figure 6). The protein abundance of MAFbx/atrogin-1 was significantly increased by 5.2-folds in the compressed muscle when compared to uncompressed muscle of DMSO-treated animals whereas no significant difference was found between compressed and uncompressed muscles in MG132-treated animals (Figure 6).

### MG132 treatment precluded compression-induced decrease in HSP70-3 mRNA expression

**Figure 7:**
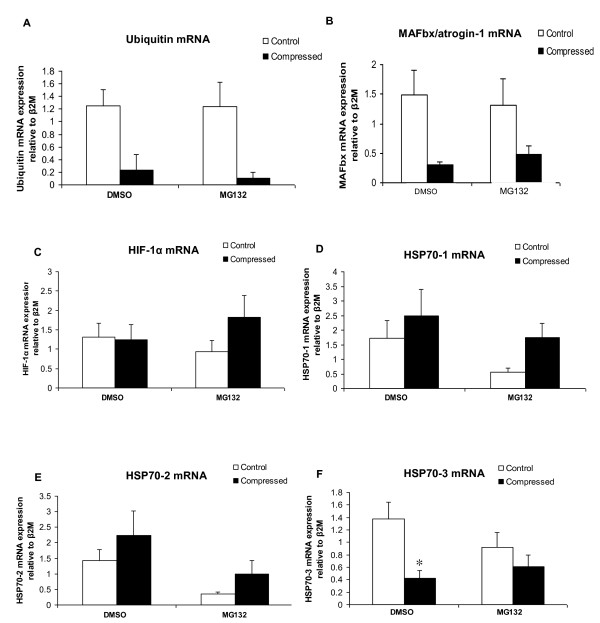
**mRNA Expression of Ubiquitin, MAFbx/atrogin-1, HIF-1α, HSP70-1, HSP70-2, and HSP70-3**. The mRNA expression levels of Ubiquitin (A), MAFbx/atrogin-1 (B), HIF-1α (C), HSP70-1 (D), HSP70-2 (E), and HSP70-3 (F) in compressed and control muscles in DMSO and MG132 treated animals were evaluated by real time PCR analysis. Data are expressed as expression ratio normalized to β2M gene and presented as mean ± SEM with significance level set at *p < 0.05, compressed muscle compared to control uncompressed muscle in the corresponding group.

According to our real time PCR analysis, the mRNA content of ubiquitin and MAFbx/atrogin-1 appeared to be decreased in the compressed muscle relative to uncompressed muscle in DMSO- and MG132-treated animals but these changes did not reach the statistical significance level (p > 0.05) (Figure 7). No significant difference was found in the mRNA abundance of HIF-1α, HSP70-1, and HSP70-2 between the compressed and uncompressed muscles in DMSO- and MG132-treated rats (Figure 7). The mRNA abundance of HSP70-3 was significantly decreased by 69% in the compressed muscle when compared to uncompressed muscle of DMSO-treated rats whereas no significant difference was found between the compressed and uncompressed muscles in MG132-treated animals (Figure 7).

### MG132 treatment precluded compression-induced increase in DNA strand breaks

**Figure 8:**
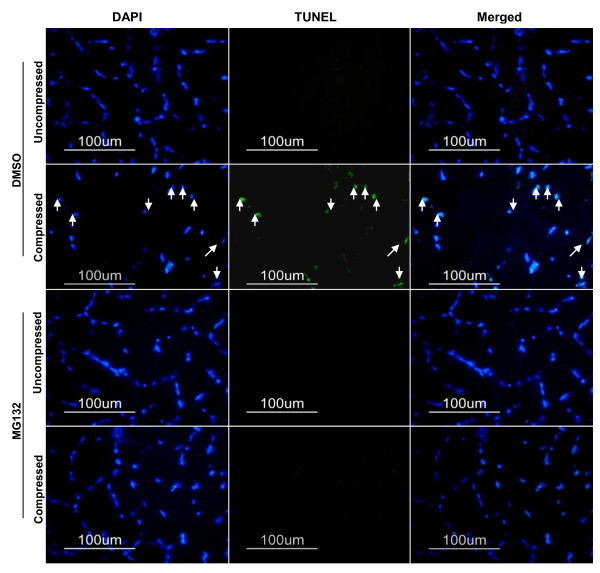
**TUNEL Analysis**. DNA strand breaks in uncompressed and compressed muscles of DMSO-treated and MG132-treated animals were assessed by TUNEL assay. TUNEL labelling was stained in green and nuclei were labelled by DAPI staining in blue. TUNEL-positive nuclei were identified in the images as shown by arrows.

TUNEL-positive nuclei were not detected in the uncompressed muscle of DMSO-treated animals whereas TUNEL-positive nuclei were observed in the compressed muscle of DMSO-treated animals. There were no TUNEL-positive nuclei detected in either the uncompressed or the compressed muscle of MG132-treated animals (Figure 8).

### MAFbx/atrogin-1 protein expression was increased immediately after compression

**Figure 9:**
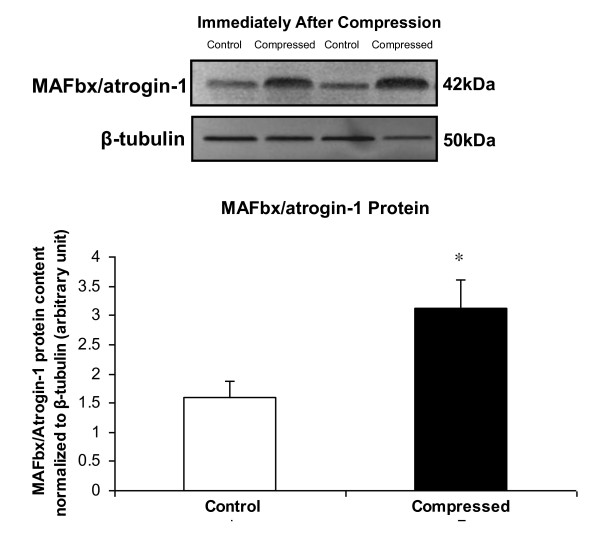
**Protein Expression of MAFbx/atrogin-1 Immediately After Compression**. The protein expression of MAFbx/atrogin-1 in muscle tissues collected immediately after the compression procedure was determined by Western blot. Data are presented as net intensity x resulting band area and expressed in arbitrary units. Result of MAFbx/atrogin-1 was normalized to corresponding β-tubulin signal. Data are presented as mean ± SEM. *p < 0.05, compressed muscle compared to uncompressed control muscle.

According to our Western blot analysis, the protein abundance of MAFbx/atrogin-1 was significantly increased by 96% in the compressed muscle of 2D-IM group (i.e., rats were sacrificed immediately after two sessions of 6-hours of compression over two consecutive days) (Figure 9).

## Discussion

Pressure-induced deep tissue injury is a severe, life-threatening form of pressure ulcer which causes considerable health concern because there are no effective treatments. There is an urgent need to understand the pathology of deep ulcer in order to identify the molecular targets for exploring new therapeutic regimens. In this study, our novel data demonstrated that the ubiquitin proteasome system is activated as indicated by the elevation of proteasome activity, ubiquitin, and the muscle-specific ubiquitin E3 ligase MAFbx/atrogin-1 in an experimental deep tissue injury protocol. Our data further demonstrated that proteasome inhibitor MG132 prevented the compression-induced increases in ubiquitin, MAFbx/atrogin-1, and 20S proteasome activity, and alleviated the muscle pathohistology induced by prolonged moderate compression. These results strongly suggested that the muscle ubiquitin proteasome system plays a role in the underlying mechanisms of deep pressure ulcer.

The ubiquitin proteasome system has been shown to function in the breakdown of muscle [36]. Ubiquitin E3 ligases are essential components in the ubiquitin proteasome system, and have been demonstrated to be expressed in increased amounts in catabolic conditions, during muscle atrophy, metabolic dysregulation, burn injury, inflammation, eccentric exercise-induced muscle damage, alcohol intoxication, and spinal cord injury [37-43]. The ubiquitin proteasome system signalling has not been investigated in pressure-induced muscle injury previously. Our immunocytochemical and biochemical data presented here illustrate that ubiquitin and MAFbx/atrogin-1 protein expression and 20S proteasome activity were elevated in response to sustained moderate compression. It is worth noting that the expression pattern of MAFbx/atrogin-1 was localized concurrently with the inflammation-associated pathohistological appearance such as the massive accumulation of nuclei in the interstitial space of muscle tissue. These observations were generally consistent with previous findings showing that MAFbx/atrogin-1 and MuRF1 were induced in response to acute inflammation as initiated by lipopolysaccharide injection and sepsis [44]. This strengthens the case that the increase in the expression of MAFbx/atrogin-1 and ubiquitin is linked to the proposed role of the ubiquitin proteasome system in the pathogenesis of deep tissue injury.

Pharmacological inhibition of the ubiquitin proteasome system using MG132 has been meticulously investigated in skeletal muscle disorders such as muscle dystrophy. Bonuccelli and colleagues have examined the effect of MG132 treatment on the mdx phenotype-related reduction of the dystrophin and dystrophin-associated proteins expression in skeletal muscle fibres from mdx mice [45]. By using the technique of immunofluorescence and Western immunoblotting, the administration of MG132, either by local injection into the muscle or osmotic minipump-mediated systemic treatment, was demonstrated to effectively rescue the expression level and sarcolemmal localization of dystrophin, β-dystroglycan, α-dystroglycan, and α-sarcoglycan in gastrocnemius muscle of mdx mice [45]. The histological analysis also illustrated the decrease in the extent of pathological changes in the muscle of mdx mice following 8-days of systemic treatment with MG132 [45]. By using a tissue explant culture model in examining the isolated skeletal muscle biopsies, MG132 treatment was further demonstrated to be successful in restoring the expression of the protein component of dystrophin-glycoprotein complex comprising dystrophin, β-dystroglycan, and α-sarcoglycan at the sarcolemma of muscles from patients with Duchenne muscular dystrophy (DMD) and Becker muscle dystrophy (BMD) [46]. Although the nature of myopathy in pressure-induced deep tissue injury is different from the dystrophin-deficiency disorders (i.e., patients with DMD/BMD and mdx mice), the present findings indicated that the inflammation-associated myopathic histology as induced by sustained compression can also be alleviated by MG132 treatment. These results were consistent with the reported protective effect of MG132 in muscle of mdx mice and DMD/BMD patients [45,46]. A notable finding refers to the alleviation of the immune cells infiltration in the compressed muscle of the MG132-treated animals. The mechanism responsible for this phenomenon is not known. It is speculated that the relationship between the ubiquitin proteasome system and immune cell signalling (e.g., NF-kappaB activation) might account for the presently observed phenomenon [11,12]. In support to the present findings, proteasome inhibitors have been demonstrated to have anti-inflammatory activities by blocking the activation of NF-kappaB and by reducing the production of inflammatory mediators and cytokines [47,48]. Furthermore, the in vivo administration of peptide aldehyde inhibitor MG132 has been shown to reduce diabetes after adoptive transfer of autoreactive T cells to nonobese diabetic mice lacking endogenous T cells and this was probably associated with the prevention of T cell proliferation [49]. Their data also illustrated that MG132 can prevent the proliferation of BDC2.5 T cells in vitro in a dose-dependent fashion [49]. Intriguingly, the effects of proteasome inhibitors on blocking the cell cycle entry of T cells and reducing the severity of some immune-mediated disorders have been demonstrated [47,50,51]. Collectively, though the exact mechanism in explaining the rescuing effect of proteasome inhibitor in deep pressure ulcer is not known, the present findings might have clinical implications in the management of deep pressure ulcer.

An intriguing finding in this study is the prevention of increased DNA strand breaks as measured by TUNEL in the MG132-treated muscle following compression. The relationship of the ubiquitin proteasome system and the regulation of apoptosis has been generally established in non-muscle and muscle cell types [52-54]. However, the inhibition of proteasome has been shown to have opposite effects in inducing and suppressing apoptosis in multiple cell types [52-54]. It has been postulated that the opposite effects of proteasome inhibition might be partly attributed to the differentiation status of the cell types [52-54]. Although the precise association of proteasome and apoptosis remains to be elucidated, our findings indicated that compression-induced muscle pathohistology accompanied by increased DNA fragmentation could be alleviated by the administration of the proteasome inhibitor MG132. Whether the observed inhibitory effect of MG132 on the compression-induced elevation of TUNEL-determined DNA fragmentation might be related to the highly differentiated postmitotic nature of skeletal muscle remains to be elucidated.

With reference to the results reported by Kwan and co-workers [29], the present study reproduced the pathologic histology in muscle tissue by adopting a similar compression protocol. It is noted that the observed pathologic characteristics were located in the zone underneath the indenter and ranged from indenter to bone as indicated by our histological analysis. It has been suggested that the tolerance and resistance to mechanical compression in muscle tissue is lower than that in soft tissues, such as cutaneous tissue [23-26]. In agreement with the suggestion that muscle tissue is more susceptible to pressure-related injury, our data showed that the pathologic characteristics were exclusively observed in the underlying muscle tissue whereas no apparent histological indication of injury was found in the skin layer (data not shown). It is noted that the present observations are consistent with the previous findings in the etiology of deep tissue injury using an animal model with controlled external loading techniques [55,56]. Those findings showed that damage was incident to the soft tissues in the zone underneath the indenter and consisted of loss of cross-striation and infiltration of macrophages [55,56]. It appears that muscle tissues directly enveloping bone surfaces are more vulnerable to pressure and resulting in pathologic change probably because of the high concentrated load and mechanical stress that the muscle needs to bear with compression. Accompanied by the demonstrated pathologic role of the ubiquitin proteasome system, the present study put forward muscle as an important initiating site for deep pressure ulcer. These findings warrant further investigation in comprehensively identifying the precise etiologic role of muscle in pressure-induced deep tissue injury.

## Conclusion

Our results demonstrated the elevation of ubiquitin, MAFbx/atrogin-1, and 20S proteasome activity in the presence of pathohistology in muscle following sustained compression in an experimental deep pressure ulcer rat model. The administration of the proteasome inhibitor MG132 was effective in preventing the pathohistology, increase in ubiquitin and MAFbx/atrogin-1, and increase in 20S proteasome activity caused by compression. Our results are consistent with the hypothesis that the muscle ubiquitin proteasome system is involved in the pathology of pressure-induced deep tissue injury. These findings suggest that the ubiquitin proteasome system could be a potential molecular target for exploring therapeutic interventions to treat pressure-induced deep tissue injury.

## Competing interests

The authors declare that they have no competing interests.

## Authors' contributions

PS participated in the design and coordination of the study, managed the data collection and drafted the manuscript. BT carried out the biochemical/molecular analysis and performed the statistical analysis. XP participated in the molecular measurement. ET conceived of the study and participated in the design of the study. All authors read and approved the final manuscript.

## Pre-publication history

The pre-publication history for this paper can be accessed here:

http://www.biomedcentral.com/1471-2474/12/58/prepub

## References

[B1] Garcia AD Thomas DR Assessment and management of chronic pressure ulcers in the elderly Med Clin North Am 2006 90 925 944 10.1016/j.mcna.2006.05.018 16962850

[B2] Cullum N McInnes E Bell-Syer SE Legood R Support surfaces for pressure ulcer prevention Cochrane Database Syst Rev 2004 CD001735 15266452 10.1002/14651858.CD001735.pub2

[B3] Gawlitta D Li W Oomens CW Baaijens FP Bader DL Bouten CV The relative contributions of compression and hypoxia to development of muscle tissue damage: an in vitro study Ann Biomed Eng 2007 35 273 284 10.1007/s10439-006-9222-5 17136445

[B4] Ankrom MA Bennett RG Sprigle S Langemo D Black JM Berlowitz DR Lyder CH Pressure-related deep tissue injury under intact skin and the current pressure ulcer staging systems Adv Skin Wound Care 2005 18 35 42 10.1097/00129334-200501000-00016 15714036

[B5] Black J Baharestani M Cuddigan J Dorner B Edsberg L Langemo D Posthauer ME Ratliff C Taler G National Pressure Ulcer Advisory Panel's updated pressure ulcer staging system Urol Nurs 2007 27 144 50 156 17494455

[B6] Donnelly J Should we include deep tissue injury in pressure ulcer staging systems? The NPUAP debate J Wound Care 2005 14 207 210 15909435 10.12968/jowc.2005.14.5.26774

[B7] Gefen A Bioengineering models of deep tissue injury Adv Skin Wound Care 2008 21 30 36 10.1097/01.ASW.0000305403.89737.6c 18156827

[B8] Fleck CA Suspected deep tissue injury Adv Skin Wound Care 2007 20 413 415 10.1097/01.ASW.0000280206.96378.5b 17620743

[B9] Gefen A Risk factors for a pressure-related deep tissue injury: a theoretical model Med Biol Eng Comput 2007 45 563 573 10.1007/s11517-007-0187-9 17486382

[B10] Arnason T Ellison MJ Stress resistance in Saccharomyces cerevisiae is strongly correlated with assembly of a novel type of multiubiquitin chain Mol Cell Biol 1994 14 7876 7883 7969127 10.1128/mcb.14.12.7876-7883.1994 PMC359326

[B11] Deng L Wang C Spencer E Yang L Braun A You J Slaughter C Pickart C Chen ZJ Activation of the IkappaB kinase complex by TRAF6 requires a dimeric ubiquitin-conjugating enzyme complex and a unique polyubiquitin chain Cell 2000 103 351 361 10.1016/S0092-8674(00)00126-4 11057907

[B12] Wertz IE O'Rourke KM Zhou H Eby M Aravind L Seshagiri S Wu P Wiesmann C Baker R Boone DL Ma A Koonin EV Dixit VM De-ubiquitination and ubiquitin ligase domains of A20 downregulate NF-kappaB signalling Nature 2004 430 694 699 10.1038/nature02794 15258597

[B13] Glickman MH Ciechanover A The ubiquitin-proteasome proteolytic pathway: destruction for the sake of construction Physiol Rev 2002 82 373 428 11917093 10.1152/physrev.00027.2001

[B14] Taillandier D Combaret L Pouch MN Samuels SE Bechet D Attaix D The role of ubiquitin-proteasome-dependent proteolysis in the remodelling of skeletal muscle Proc Nutr Soc 2004 63 357 361 10.1079/PAR2004358 15294055

[B15] Zhang P Chen X Fan M Signaling mechanisms involved in disuse muscle atrophy Med Hypotheses 2007 69 310 321 10.1016/j.mehy.2006.11.043 17376604

[B16] Bodine SC Latres E Baumhueter S Lai VK Nunez L Clarke BA Poueymirou WT Panaro FJ Na E Dharmarajan K Pan ZQ Valenzuela DM DeChiara TM Stitt TN Yancopoulos GD Glass DJ Identification of ubiquitin ligases required for skeletal muscle atrophy Science 2001 294 1704 1708 10.1126/science.1065874 11679633

[B17] Gomes MD Lecker SH Jagoe RT Navon A Goldberg AL Atrogin-1, a muscle-specific F-box protein highly expressed during muscle atrophy Proc Natl Acad Sci USA 2001 98 14440 14445 10.1073/pnas.251541198 11717410 PMC64700

[B18] Franch HA Price SR Molecular signaling pathways regulating muscle proteolysis during atrophy Curr Opin Clin Nutr Metab Care 2005 8 271 275 10.1097/01.mco.0000165005.01331.45 15809529

[B19] Miyazaki M Noguchi M Takemasa T Intermittent reloading attenuates muscle atrophy through modulating Akt/mTOR pathway Med Sci Sports Exerc 2008 40 848 855 10.1249/MSS.0b013e318163275f 18408614

[B20] de Palma L Marinelli M Pavan M Orazi A Ubiquitin ligases MuRF1 and MAFbx in human skeletal muscle atrophy Joint Bone Spine 2008 75 53 57 10.1016/j.jbspin.2007.04.019 17977773

[B21] Edlich RF Winters KL Woodard CR Buschbacher RM Long WB Gebhart JH Ma EK Pressure ulcer prevention J Long Term Eff Med Implants 2004 14 285 304 10.1615/JLongTermEffMedImplants.v14.i4.20 15447627

[B22] Struck BD Wright JE Pressure ulcers and endothelial dysfunction: is there a link? J Nutr Elder 2007 26 105 117 10.1300/J052v26n03_05 18285294

[B23] Bouten CV Oomens CW Baaijens FP Bader DL The etiology of pressure ulcers: skin deep or muscle bound? Arch Phys Med Rehabil 2003 84 616 619 10.1053/apmr.2003.50038 12690603

[B24] Daniel RK Priest DL Wheatley DC Etiologic factors in pressure sores: an experimental model Arch Phys Med Rehabil 1981 62 492 498 7305643

[B25] Nola GT Vistnes LM Differential response of skin and muscle in the experimental production of pressure sores Plast Reconstr Surg 1980 66 728 733 10.1097/00006534-198011000-00008 7001518

[B26] Salcido R Donofrio JC Fisher SB LeGrand EK Dickey K Carney JM Schosser R Liang R Histopathology of pressure ulcers as a result of sequential computer-controlled pressure sessions in a fuzzy rat model Adv Wound Care 1994 7 23 4 26, 28 7889250

[B27] Attaix D Ventadour S Codran A Bechet D Taillandier D Combaret L The ubiquitin-proteasome system and skeletal muscle wasting Essays Biochem 2005 41 173 186 10.1042/EB0410173 16250905

[B28] Cao PR Kim HJ Lecker SH Ubiquitin-protein ligases in muscle wasting Int J Biochem Cell Biol 2005 37 2088 2097 10.1016/j.biocel.2004.11.010 16125112

[B29] Kwan MP Tam EW Lo SC Leung MC Lau RY The time effect of pressure on tissue viability: investigation using an experimental rat model Exp Biol Med (Maywood) 2007 232 481 487 17392483

[B30] Siu PM Tam EW Teng BT Pei XM Ng JW Benzie IF Mak AF Muscle apoptosis is induced in pressure-induced deep tissue injury J Appl Physiol 2009 107 1266 1275 10.1152/japplphysiol.90897.2008 19644027

[B31] Teng BT Pei XM Tam EW Benzie IF Siu PM Opposing responses of apoptosis and autophagy to moderate compression in skeletal muscle Acta Physiol (Oxf) 2010 201 239 254 10.1111/j.1748-1716.2010.02173.x 20670304

[B32] Rock KL Gramm C Rothstein L Clark K Stein R Dick L Hwang D Goldberg AL Inhibitors of the proteasome block the degradation of most cell proteins and the generation of peptides presented on MHC class I molecules Cell 1994 78 761 771 10.1016/S0092-8674(94)90462-6 8087844

[B33] Chen X Li SL Wu T Liu JD Proteasome inhibitor ameliorates severe acute pancreatitis and associated lung injury of rats World J Gastroenterol 2008 14 3249 3253 10.3748/wjg.14.3249 18506934 PMC2712861

[B34] Yuan B Xing Y Horst RL Drezner MK Evidence for abnormal translational regulation of renal 25-hydroxyvitamin D-1alpha-hydroxylase activity in the hyp-mouse Endocrinology 2004 145 3804 3812 10.1210/en.2004-0192 15265826

[B35] Livak KJ Schmittgen TD Analysis of relative gene expression data using real-time quantitative PCR and the 2(-Delta Delta C(T)) Method Methods 2001 25 402 408 10.1006/meth.2001.1262 11846609

[B36] Mitch WE Goldberg AL Mechanisms of muscle wasting. The role of the ubiquitin-proteasome pathway N Engl J Med 1996 335 1897 1905 10.1056/NEJM199612193352507 8948566

[B37] Granado M Martin AI Priego T Lopez-Calderon A Villanua MA Tumour necrosis factor blockade did not prevent the increase of muscular muscle RING finger-1 and muscle atrophy F-box in arthritic rats J Endocrinol 2006 191 319 326 10.1677/joe.1.06931 17065414

[B38] Nedergaard A Vissing K Overgaard K Kjaer M Schjerling P Expression patterns of atrogenic and ubiquitin proteasome component genes with exercise: effect of different loading patterns and repeated exercise bouts J Appl Physiol 2007 103 1513 1522 10.1152/japplphysiol.01445.2006 17690190

[B39] Witt CC Witt SH Lerche S Labeit D Back W Labeit S Cooperative control of striated muscle mass and metabolism by MuRF1 and MuRF2 EMBO J 2008 27 350 360 10.1038/sj.emboj.7601952 18157088 PMC2168395

[B40] Drummond MJ Glynn EL Lujan HL Dicarlo SE Rasmussen BB Gene and protein expression associated with protein synthesis and breakdown in paraplegic skeletal muscle Muscle Nerve 2008 37 505 513 10.1002/mus.20976 18236467 PMC2715297

[B41] Koyama S Hata S Witt CC Ono Y Lerche S Ojima K Chiba T Doi N Kitamura F Tanaka K Abe K Witt SH Rybin V Gasch A Franz T Labeit S Sorimachi H Muscle RING-finger protein-1 (MuRF1) as a connector of muscle energy metabolism and protein synthesis J Mol Biol 2008 376 1224 1236 10.1016/j.jmb.2007.11.049 18222470

[B42] Lang CH Huber D Frost RA Burn-induced increase in atrogin-1 and MuRF-1 in skeletal muscle is glucocorticoid independent but downregulated by IGF-I Am J Physiol Regul Integr Comp Physiol 2007 292 R328 R336 16946078 10.1152/ajpregu.00561.2006

[B43] Vary TC Frost RA Lang CH Acute alcohol intoxication increases atrogin-1 and MuRF1 mRNA without increasing proteolysis in skeletal muscle Am J Physiol Regul Integr Comp Physiol 2008 294 R1777 R1789 18401005 10.1152/ajpregu.00056.2008 PMC3585419

[B44] Dehoux MJ van Beneden RP Fernandez-Celemin L Lause PL Thissen JP Induction of MafBx and Murf ubiquitin ligase mRNAs in rat skeletal muscle after LPS injection FEBS Lett 2003 544 214 217 10.1016/S0014-5793(03)00505-2 12782319

[B45] Bonuccelli G Sotgia F Schubert W Park DS Frank PG Woodman SE Insabato L Cammer M Minetti C Lisanti MP Proteasome inhibitor (MG-132) treatment of mdx mice rescues the expression and membrane localization of dystrophin and dystrophin-associated proteins Am J Pathol 2003 163 1663 1675 10.1016/S0002-9440(10)63523-7 14507673 PMC1868305

[B46] Assereto S Stringara S Sotgia F Bonuccelli G Broccolini A Pedemonte M Traverso M Biancheri R Zara F Bruno C Lisanti MP Minetti C Pharmacological rescue of the dystrophin-glycoprotein complex in Duchenne and Becker skeletal muscle explants by proteasome inhibitor treatment Am J Physiol Cell Physiol 2006 290 C577 C582 10.1152/ajpcell.00434.2005 16192300

[B47] Luo H Wu Y Qi S Wan X Chen H Wu J A proteasome inhibitor effectively prevents mouse heart allograft rejection Transplantation 2001 72 196 202 10.1097/00007890-200107270-00005 11477338

[B48] Meng L Mohan R Kwok BH Elofsson M Sin N Crews CM Epoxomicin, a potent and selective proteasome inhibitor, exhibits in vivo antiinflammatory activity Proc Natl Acad Sci USA 1999 96 10403 10408 10.1073/pnas.96.18.10403 10468620 PMC17900

[B49] Petrovic J Hall H Mehr R Glas R Hoglund P Inhibition of the proteasome reduces transfer-induced diabetes in nonobese diabetic mice Scand J Immunol 2004 60 134 142 10.1111/j.0300-9475.2004.01473.x 15238082

[B50] Wang X Luo H Chen H Duguid W Wu J Role of proteasomes in T cell activation and proliferation J Immunol 1998 160 788 801 9551914

[B51] Zollner TM Podda M Pien C Elliott PJ Kaufmann R Boehncke WH Proteasome inhibition reduces superantigen-mediated T cell activation and the severity of psoriasis in a SCID-hu model J Clin Invest 2002 109 671 679 11877475 10.1172/JCI12736 PMC150886

[B52] Wojcik C Regulation of apoptosis by the ubiquitin and proteasome pathway J Cell Mol Med 2002 6 25 48 10.1111/j.1582-4934.2002.tb00309.x 12003667 PMC6740140

[B53] Drexler HC Programmed cell death and the proteasome Apoptosis 1998 3 1 7 10.1023/A:1009604900979 14646512

[B54] Orlowski RZ The role of the ubiquitin-proteasome pathway in apoptosis Cell Death Differ 1999 6 303 313 10.1038/sj.cdd.4400505 10381632

[B55] Bosboom EM Bouten CV Oomens CW van Straaten HW Baaijens FP Kuipers H Quantification and localisation of damage in rat muscles after controlled loading; a new approach to study the aetiology of pressure sores Med Eng Phys 2001 23 195 200 10.1016/S1350-4533(01)00034-0 11410384

[B56] Stekelenburg A Oomens CW Strijkers GJ Nicolay K Bader DL Compression-induced deep tissue injury examined with magnetic resonance imaging and histology J Appl Physiol 2006 100 1946 1954 10.1152/japplphysiol.00889.2005 16484364

